# The Oncogene IARS2 Promotes Non-small Cell Lung Cancer Tumorigenesis by Activating the AKT/MTOR Pathway

**DOI:** 10.3389/fonc.2019.00393

**Published:** 2019-05-14

**Authors:** Xin Di, Xin Jin, He Ma, Ruimin Wang, Shan Cong, Chang Tian, Jiaying Liu, Min Zhao, Ranwei Li, Ke Wang

**Affiliations:** ^1^Department of Respiratory Medicine, The Second Hospital of Jilin University, Changchun, China; ^2^Department of Oncology and Hematology, The Second Hospital of Jilin University, Changchun, China; ^3^Department of Anesthesiology, The Second Hospital of Jilin University, Changchun, China; ^4^Department of Operation Room, The Second Hospital of Jilin University, Changchun, China; ^5^Department of Urinary Surgery, The Second Hospital of Jilin University, Changchun, China

**Keywords:** isoleucyl-tRNA synthetase 2, lung cancer, tumorigenesis, cDNA microarray, ingenuity pathway analysis, AKT, mammalian target of rapamycin

## Abstract

A limited number of studies have indicated an association between isoleucyl-tRNA synthetase 2 (IARS2) and tumorigenesis. We evaluated IARS2 protein expression in lung tumor tissues and paired non-tumor tissues. We found higher IARS2 expression in the tumor tissues, which was associated with the late Tumor and Node stages of the Tumor, Node, Metastasis staging system. Silencing IARS2 inhibited the activity of A549 and H1299 cells, resulting in G0/G1 stasis of A549 cells and mitochondrial apoptosis. IARS2 silencing was also found to inhibit NSCLC tumor growth in nude mice. Complementary DNA microarray analysis revealed 742 differentially expressed genes (507 upregulated and 235 downregulated) in IARS2-silenced A549 cells compared to controls. Ingenuity Pathway Analysis of the differential expression data suggested that multiple pathways are associated with IARS2 silencing in NSCLC cells; upstream analysis predicted the activation or inhibition of transcriptional regulators. Correlation analysis revealed that AKT and MTOR activities were significantly inhibited in IARS2-silenced cells, but were partially restored by the AKT-stimulating agent SC79. IARS2 appears to regulate lung cancer cell proliferation via the AKT/MTOR pathway. Our results help clarify the complex roles of IARS2 in tumorigenesis and suggest that it may be a novel regulator of lung cancer development.

## Introduction

Lung cancer remains the most malignant tumor with the highest morbidity and mortality worldwide; for 2018, 2.1 million new lung cancer cases and 1.8 million deaths have been predicted, representing nearly 1 in 5 (18.4%) cancer deaths (1). Lung cancer is commonly classified into small cell carcinoma and non-small cell lung carcinoma (NSCLC) (2). In most countries, patients with adenocarcinoma are more common than those with squamous cell carcinoma (3). Active exploration of the pathogenesis of lung cancer has indicated that the identification of new lung cancer-related biomarkers may be crucial to improve the treatment and prognosis of patients with lung cancer.

The aminoacyl-tRNA synthetase (ARSs) class of evolutionarily ancient enzymes is widely found in organisms. ARSs are responsible for catalysing the esterification of the hydroxyl group of tRNAs with the carboxyl group of the corresponding amino acids to form aminoacyl tRNAs (4). Mammalian ARSs have evolved many new non-catalytic domains to perform non-canonical functions (5). ARSs play important roles in tumor pathogenesis by regulating tumor cell growth, differentiation, cell cycle, cytokine activity, RNA splicing, cell adhesion, and angiogenesis (6–8).

Isoleucyl-tRNA synthetase 2, mitochondrial (IARS2) is a nuclear gene encoding a mitochondrial ARS. The expression of IARS2 mRNA in human colon cancer tissues is higher than that in surrounding tissues. Knocking down the IARS2 gene inhibits the proliferation of colon cancer RKO cells, increases the proportion of cells in G0/G1 phase, and decreases the proportion of cells in S phase (9). In gastric cancer AGS cells, IARS2 knockdown inhibits proliferation and colony formation and induces cell cycle arrest in the G2/M phase (10). The expression of IARS2 in short-term survivors of glioblastoma is higher than that in long-term survivors, suggesting that high IARS2 expression is a risk factor for glioblastoma (11). These results suggest that IARS2 may be involved in the development and progression of tumors. However, the role of IARS2 in the development of NSCLC and its related molecular mechanisms are not well defined.

## Materials and Methods

### Tissue Specimens

We enrolled 56 patients with primary NSCLC who underwent surgery at the Second Hospital of Jilin University from May 2017 to August 2018. The patients had not received chemotherapy or radiation before surgery. All patients were diagnosed according to the World Health Organization's lung cancer criteria and staged according to the Tumor (T), Node (N), Metastasis (M) staging system for lung cancer (12). This study was approved by the Ethics Committee of the Second Hospital of Jilin University (Changchun, China) and all participants provided written informed consent. Lung cancer and corresponding tumor-adjacent lung tissue samples were collected during surgery and stored at −80°C. All healthy and cancerous tissues were re-evaluated by pathologists.

### Cell Culture and Reagents

Human embryonic kidney 293 (HEK-293) cells and the human lung cell lines A549 and H1299 were obtained from the Chinese Academy of Medical Sciences (Beijing, China) and cultured in Dulbecco's modified Eagle's medium or RPMI-1640 (Gibco, Carlsbad, CA, USA) supplemented with 10% fetal bovine serum at 37°C in a humidified atmosphere containing 5% CO2. SC79 (catalog number S1023) was obtained from MedChemExpress (Monmouth Junction, NJ, USA).

### Plasmids, Lentiviral Production, and Transduction

The IARS2 shRNA (shIARS2) lentiviral gene transfer vector pGCSIL-GFP, which encodes the enhanced green fluorescent protein sequence, was constructed by GeneChem (Shanghai, China). The hairpin sequence of shIARS2-1 was CCGGGTACTTGCAGTCATCCATTAATTCAAGAGATTAATGGATGACTGCAAGTACTTTTTG and the sequence of shIARS2-2 was CCGGGCTTAGGAATACACTTCGCTTCTCGAGAAGCGAAGTGTATTCCTAAGCTTTTT (GenBank accession number: NM_018060). The resulting constructs were verified by sequencing. A corresponding random shRNA sequence was used as a control for shIARS2. The vectors were transfected into HEK-293 cells using Lipofectamine 2000 (Invitrogen, Carlsbad, CA, USA) according to the manufacturer's instructions. Cell culture medium containing lentiviral particles was harvested 48 h post-transfection and passed through a 0.45-μm filter (Merck Millipore, Burlington, MA, USA). The resulting lentiviral particles were stored at −80°C until use. After transfection with lentivirus for 48 h, cells were screened in puromycin culture medium to establish cell lines with a stable knockout of the IARS2 gene.

### Colony Formation Assay

Lentiviral vector-transduced A549 and H1299 cells were seeded in 6-well plates (400 cells/well). After 14 days, colonies (>50 cells/colony) were counted and individually imaged after staining with Giemsa (Beijing Solarbio Science & Technology, Beijing, China).

### Cell Counting Kit-8 Assay

We determined the viability of A549 and H1299 cells using a Cell Counting Kit-8 (CCK-8) assay (Dojindo, Tokyo, Japan). Cells (2 × 10^3^ cells/well) were seeded in 96-well plates and incubated at 37°C for 24 h. We added CCK-8 reagent (10 μL) to each well and incubated the plates for a further 1 h at 37°C. The optical density of each well was measured using a microplate reader (Thermo Fisher Scientific, Waltham, MA, USA) at a test wavelength of 450 nm.

### Flow Cytometry Analysis of Cell Cycle and Apoptosis

For cell cycle analysis, the lentiviral vector-transduced A549 and H1299 cells were labeled with propidium iodide (BD Biosciences, San Jose, CA, USA) and analyzed using flow cytometry. For apoptosis analysis, the cells were incubated with PE-conjugated annexin V and 7-AAD (BD Biosciences, San Jose, CA, USA), according to the manufacturer's guidelines, prior to flow cytometry.

### cDNA Microarray Assay and Ingenuity Pathway Analysis

To profile the expression of IARS2-regulated genes, we first stably transduced the A549 cells with the lentiviral shIARS2-1 or control vector. The cells were subjected to RNA isolation. The RNA was quantified using a NanoDrop 2000 (Thermo Fisher Scientific, Waltham, MA, USA) and checked for quality with an Agilent Bioanalyzer 2100 (Agilent Technologies, Santa Clara, CA, USA). Then, the quality-checked RNA samples were labeled with the GeneChip 3′ IVT Express Kit (Affymetrix, Santa Clara, CA, USA) and hybridized to an Affymetrix GeneChip PrimeView Human Gene Expression Array, according to the manufacturer's protocols. The experiments were conducted by GeneChem (Shanghai, China). The data were analyzed using a GeneChip Scanner 3000 (Affymetrix). We set the thresholds to determine gene alterations after IARS2 silencing at a 2.0-fold change in expression and *p* < 0.05 (false discovery rate [FDR] < 0.05) vs. the control cells. Ingenuity Pathway Analysis (IPA; Ingenuity Systems; www.ingenuity.com; Redwood City, CA, USA) is an online software package used to identify canonical pathways and gene networks and to categorize specific physiological processes. The Ingenuity Pathway Knowledge Base was used for deep analysis of the global molecular network and discovery of interactions among the differentially expressed genes.

### Western Blotting

We obtained cellular proteins by lysing lung tissue with radioimmunoprecipitation assay buffer supplemented with protease inhibitors and phosphatase inhibitors. We determined the protein concentrations using a bicinchoninic acid protein assay kit (Beyotime, Jiangsu, China) according to the instructions. Lysis proteins (30 μg) were separated by electrophoresis on 8–15% sodium dodecyl sulfate–polyacrylamide gels. We transferred the samples to polyvinylidene difluoride membranes(#PIVH00010, Merck Millipore, Burlington, MA, USA). The membranes were probed with primary antibodies and were then incubated with horseradish peroxidase-conjugated secondary antibodies. We purchased the antibodies against AKT (# 4691), phospho-AKT (S473; # 4060), phospho-AKT (T308; # 13038), BCL-2 (# 2872), BAX (# 5023), cleaved caspase 3 (# 9664), cleaved PARP (# 5625), caspase 9 (# 9508), mammalian target of rapamycin (MTOR; # 2983), and phospho-MTOR (Ser2448; # 5536) from Cell Signaling Technologies (Danvers, MA, USA). We purchased the antibodies against β-actin (# 60008-1-Ig) and IARS2 (# 17170-1-AP) from Proteintech (Rosemont, IL, USA). The signals were detected using an enhanced chemiluminescence detection kit (Merck Millipore, Burlington, MA, USA). Densitometric analysis was performed with ImageJ; relative values are displayed under their respective blots.

### Animal Studies

Five-week-old BALB/c nude mice were maintained under specific-pathogen-free conditions. We subcutaneously injected A549 cells (5 × 10^6^ cells; 2.5 × 10^7^ cell/mL in 0.2 mL phosphate-buffered saline) that stably expressed shIARS2-1, shIARS2-2, or the negative control shRNA into the left flanks of the mice. We measured the tumors with electronic calipers and calculated the sizes with the formula: volume = length × width^2^ × 0.5. Animal experiments were approved by the Institutional Animal Care and Use Committee of Jilin University.

### Statistical Analysis

Pearson's Chi-squared test was performed to determine the association of clinicopathological data with the expression of IARS2 proteins in NSCLC tissues. Statistical data are expressed as the mean ± standard deviation. Comparisons among groups were carried out with Student's *t*-test. A *p* < 0.05 was considered statistically significant. All data were analyzed using SPSS 19.0 software.

## Results

### IARS2 Expression Was Higher in NSCLC Tissues Than in Non-cancerous Controls

**Figure 1:**
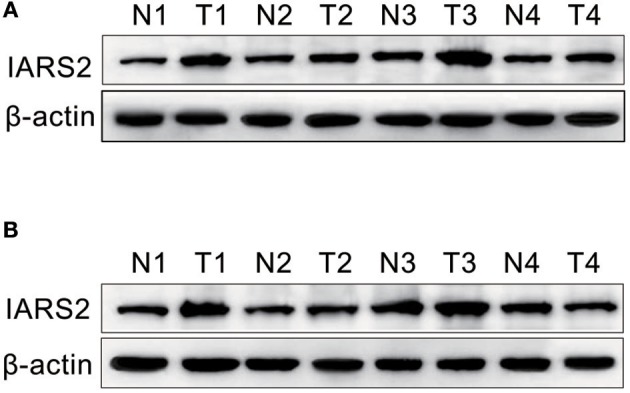
Western blotting of IARS2 protein expression in representative tissue samples from NSCLC (T) and non-tumor specimens (N). Total protein was extracted, subjected to western blotting analysis, and quantified using Image J software. **(A)** Squamous cell carcinoma tissue. **(B)** Adenocarcinoma tissue.

We measured IARS2 protein expression in 56 pairs of NSCLC and adjacent normal tissue samples. IARS2 expression was higher in NSCLC tissues, and the high expression rate of adenocarcinoma accounted for 72.73%, which was slightly higher than that of squamous cell carcinoma. Elevated IARS2 protein levels were associated with the T and N stages of advanced tumors, but not with gender, age, or smoking status (Figures 1A,B and Table 1).

**Table 1:** Association of IARS2 with clinicopathological characteristics from 56 lung cancer patients.

**Characteristic**	***N* (%)**	**IARS2 expression level**, ***N*** **(%)**		***P*-value**
		**High expression**	**Low expression**	
Histological types			
Squamous cell	34 (60.71)	22 (64.71)	12 (35.29)	0.53
Adenocarcinoma	22 (39.29)	16 (72.73)	6 (27.27)	
Age (years)			
≤ 60	24 (42.86)	18 (75.00)	6 (25.00)	0.322
>60	32 (57.14)	20 (62.50)	12 (37.50)	
Gender			
Male	41 (73.21)	27 (65.85)	14 (34.15)	0.596
Female	15 (26.79)	11 (73.33)	4 (26.67)	
Smoke			
NO	30 (53.57)	21 (70.00)	9 (30.00)	0.712
YES	26 (46.43)	17 (65.38)	9 (34.62)	
pT status			
T1–T2	34 (60.71)	19 (55.88)	15 (44.12)	**0.036**
T3–T4	22 (39.29)	19 (86.36)	3 (13.64)	
pN status			
pN–	26 (46.43)	14 (53.85)	10 (32.26)	**0.037**
pN+	30 (53.57)	24 (80.00)	8 (32.00)	

*p-values represent Pearson χ^2^ test*.

*pT status, Tumor; pN status, Node*.

*Bold values indicate P < 0.05*.

### IARS2 Knockdown Inhibited Lung Cancer Cell Viability and Colony Formation

**Figure 2:**
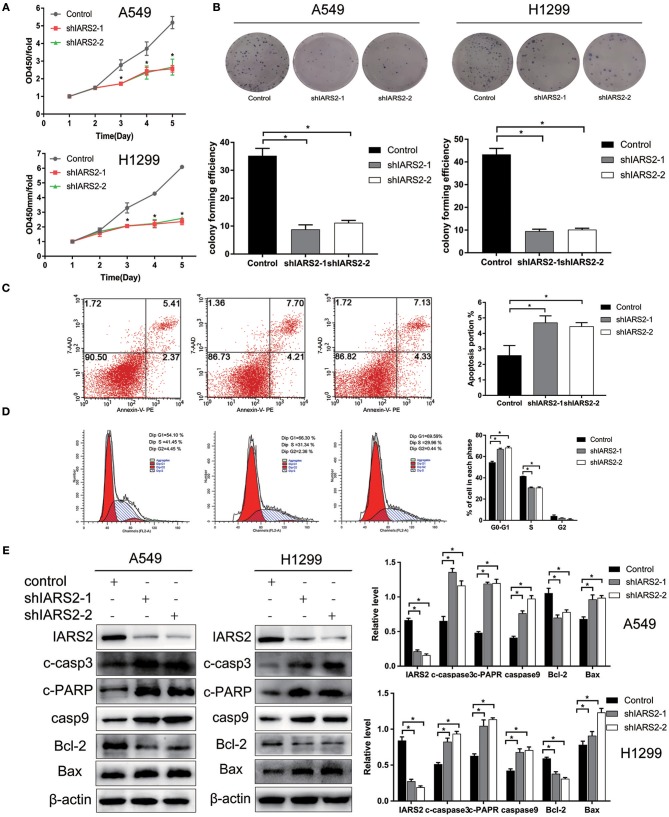
Effects of IARS2 knockdown on cell proliferation, cell cycle progression and apoptosis in NSCLC cells. **(A)** Cell Counting Kit-8 assay was used to evaluate A549 and H1299 cell growth after IARS2 knockdown. **(B)** Colony formation assay was used to evaluate A549 and H1299 cell growth after IARS2 knockdown. **(C)** Apoptosis was evaluated using flow cytometry in IARS2 knockdown and control A549 cells. Representative flow-cytograms are shown, and apoptotic rates were derived as percentages of Annexin V-PE positive cells. **(D)** Cell cycle was assessed in A549 cells using flow cytometry after transfection with the indicated shRNAs. Representative flow-cytograms are shown, as well as diagrams quantifying cell fractions in the G0/G1, S, and G2/M phases. **(E)** Differential expression of apoptosis regulatory proteins associated with IARS2 knockdown. Western blotting analysis was performed to compare expression levels of various apoptosis-related proteins between the IARS2 and control groups. ^*^p < 0.05 compared with the control group.

We stably silenced IARS2 in A549 and H1299 cells. Compared with cells in the control group, cell proliferation activity of the shIARS2-1 and the shIARS2-2 groups decreased significantly (Figure 2A). Compared with control cells expressing the empty lentiviral expression vector, the shIARS2-1 and the shIARS2-2 groups had a lower clonogenic activity (Figure 2B).

### Effect of IARS2 on Apoptosis and Cell Cycle

IARS2 silencing inhibited proliferation and promoted apoptosis of A549 cell (Figure 2C). In addition, compared to control cells, the proportion of G1 cells increased and that of S cells decreased (Figure 2D).

### Analysis of Mitochondrial Apoptotic Pathway-Related Proteins in IARS2 Mediated-Apoptosis

To further illustrate the role of mitochondrial apoptosis as a downstream molecular mechanism of IARS2, we show that the expression of cleaved caspase 3, cleaved PARP, cleaved caspase 9, and BAX increased, whereas the expression of BCL-2 decreased, in IARS2-silenced cells. This suggests that IARS2-mediated growth suppression, at least in part, occurs via modulation of the mitochondrial apoptotic pathways (Figure 2E).

### IARS2-Regulated Gene Expression Profiling

**Figure 3:**
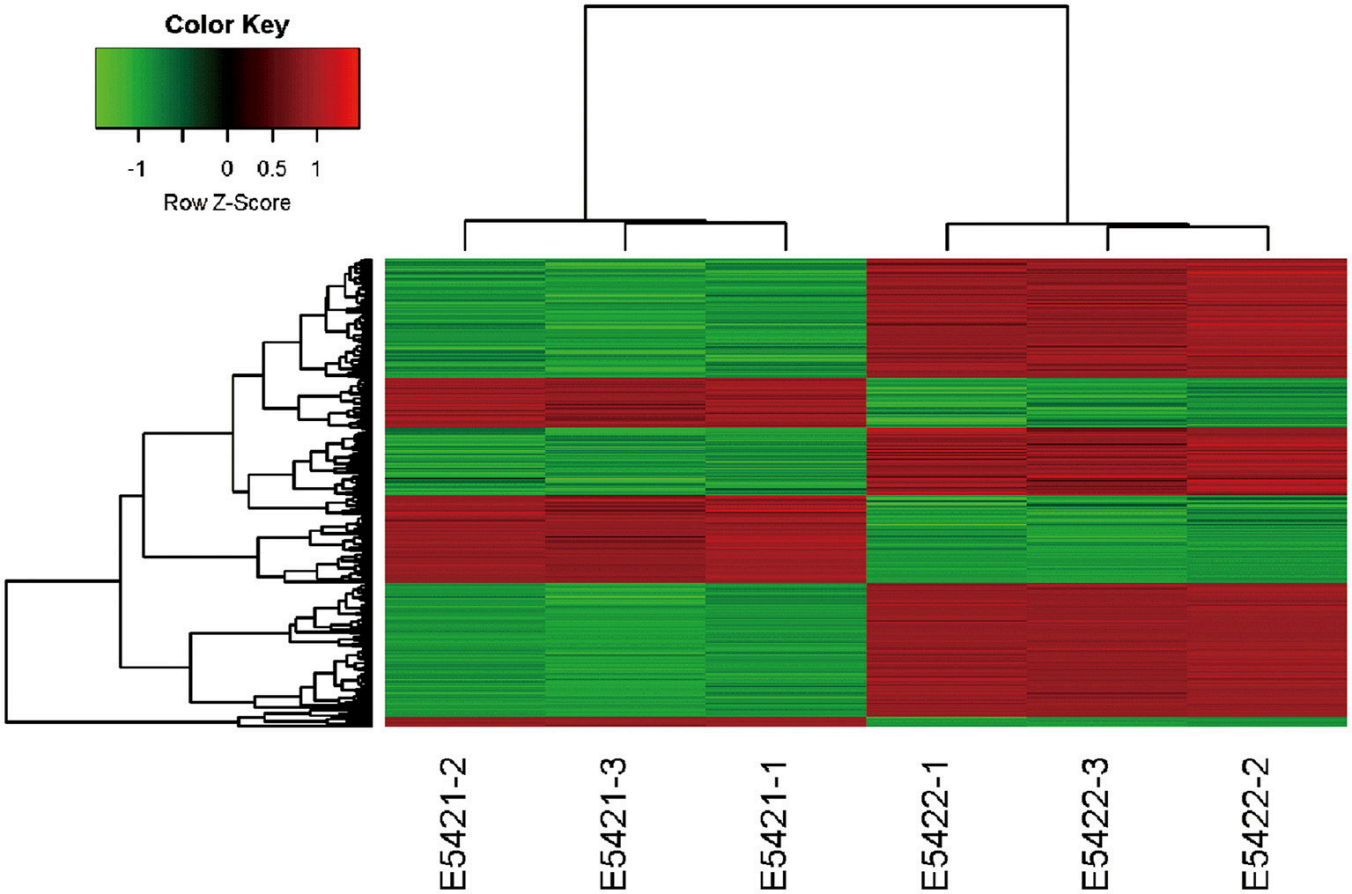
Heat map of the 2-way hierarchical clustering of genes and samples. Each row represents a gene and each column represents a sample. The gene clustering tree is shown on the left and the sample clustering tree appears at the top. Red, up-regulated in the IARS2-silenced vs. control cells; green down-regulated in the IARS2-silenced vs. control cells.

An Affymetrix GeneChip PrimeView Human cDNA microarray analysis was performed to profile the expression of IARS2-regulated genes in A549 cells (GEO accession number is GSE130007). IARS2 expression regulated the expression of 742 genes in A549 cells, of which 507 were upregulated and 235 were downregulated upon IARS2 silencing (Figure 3).

### Functional Analysis of Differentially Expressed Genes Relative to Classical Pathways, Upstream Regulators, and Disease

**Figure 4:**
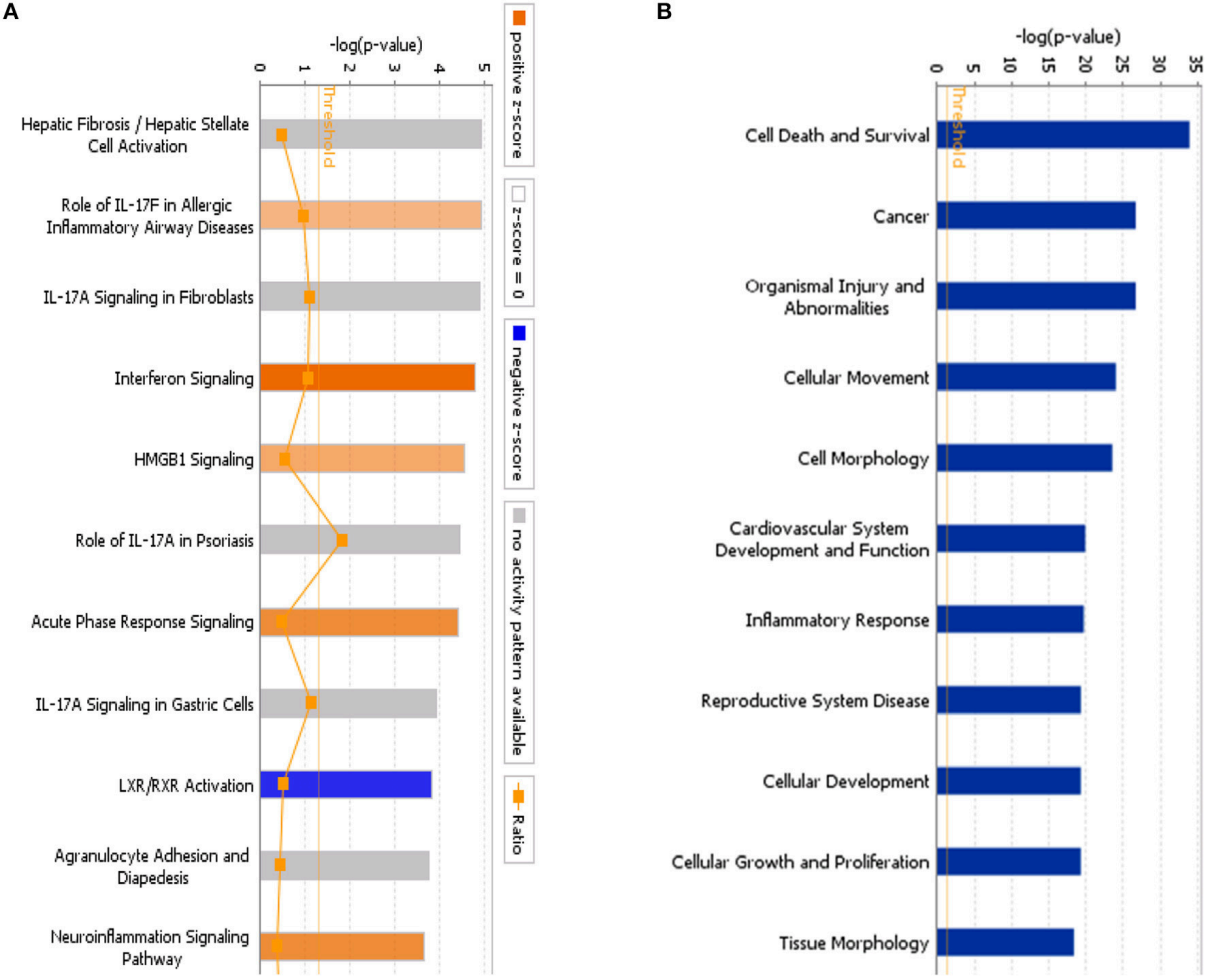
**(A)** Classical pathway enrichment analysis. The orange label indicates pathway activation (*z*-score >0), the blue label indicates pathway suppression (*z*-score < 0), and the shades of orange and blue indicate the degree of activation or inhibition (the absolute value of the *z*-score). The ratio represents the number of differentially expressed genes in this signaling pathway and the number of all genes in the pathway. **(B)** Disease and functional enrichment analysis statistics. This figure shows the differentially expressed genes in the IARS2-silenced cells that are significantly enriched in disease and function. The abscissa is the name of the path and the ordinate is the level of significance of the enrichment (the negative logarithm of the base 10).

Using IPA, we examined the relationships between the differentially expressed genes and canonical pathways. Our analysis revealed a highly significant overlap of the differentially regulated genes with 353 canonical pathways connected with apoptosis, cancer, cell cycle regulation, cellular immune responses, cellular growth, proliferation, and development. Figure 4A shows the 41 signaling pathways with the highest levels of significance.

**Figure 5:**
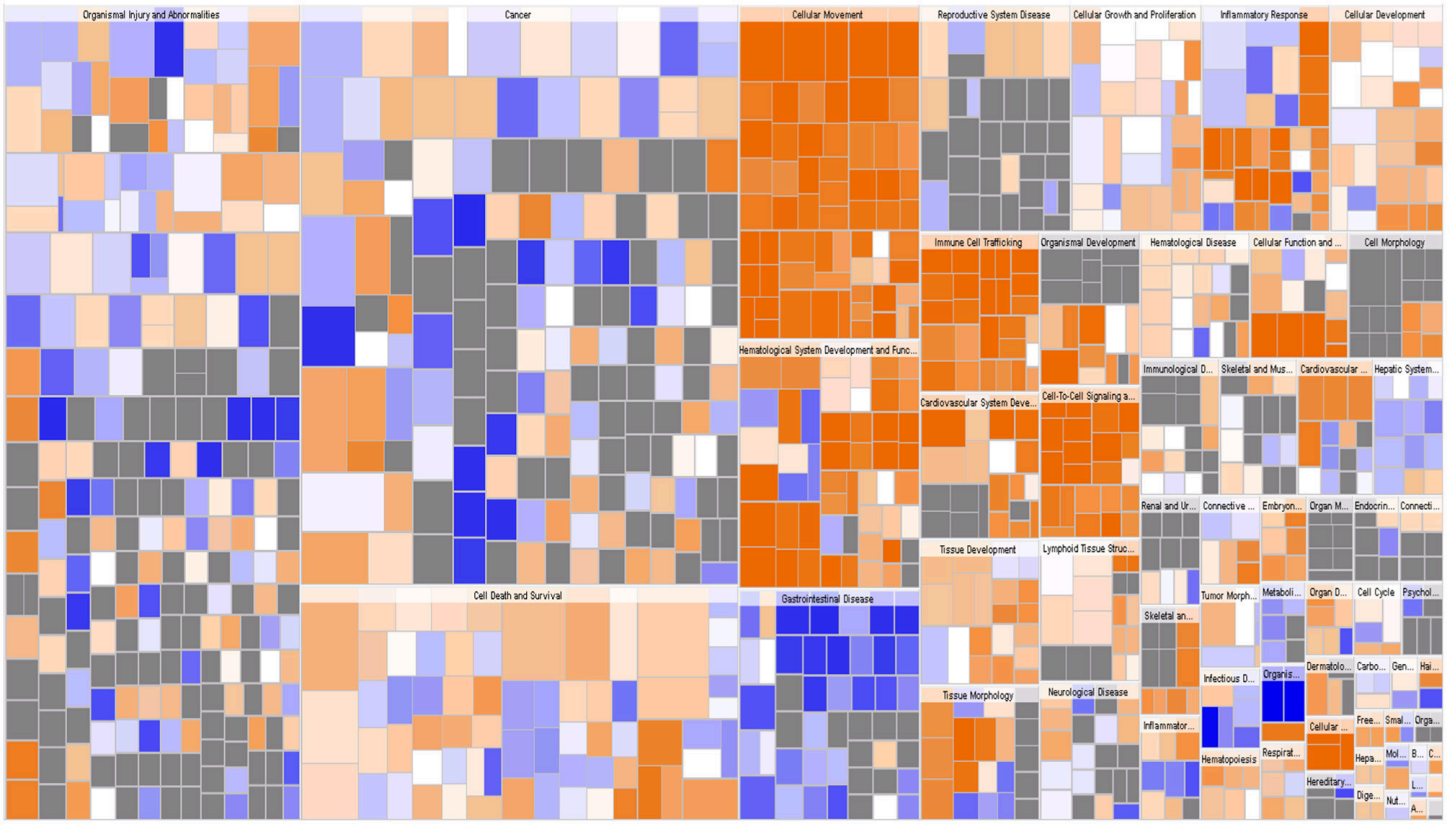
Disease and function heat map. This figure demonstrates the relationships between changes in differentially expressed gene levels and the activation and inhibition of diseases and functions. Orange indicates that the disease or functional status is activated (*z*-score >0), blue indicates that the disease or functional status is suppressed (*z*-score < 0), and gray indicates that the disease or functional status was not determined (*z*-score cannot be calculated).

IPA identified 259 diseases or functions predicted to be activated upon IARS2 silencing, of which the top 5 were cell migration, cell movement, leukocyte migration, homing of cells, and chemotaxis. Of the 132 diseases or functions that were predicted to be inhibited, the top 5 were intestinal cancer, gastrointestinal tract cancer, infection of mammalia, morbidity or mortality, and organismal death (Figures 4B, 5).

IPA uses the activation z-score algorithm to predict the activation or suppression of upstream regulators, reduce the instance of significant predictions resulting from random data, and analyse the relationships of genes to disease and function. In this study, 951 molecules (including transcription factors, cytokines, small RNAs, receptors, kinases, chemical molecules, and drugs) were predicted to be activators and 483 molecules were predicted to be inhibitors. Table 2 shows the IPA-predicted upstream activation or inhibitory molecules acting on the IARS2 gene (top 10).

**Table 2:** Comparison of the upstream regulators of IARS2.

**Upstream regulator**	**Entrez gene name**	**Predicted state**	***z*-score**	***P*-value**
TNF	Tumor necrosis factor	Activated	8.273	1.07E-43
Lipopolysaccharide	Lipopolysaccharide	Activated	8.092	8.72E-33
NFκB (complex)	Nuclear factor kappa B	Activated	6.869	3.08E-25
IL1B	Interleukin 1 Beta	Activated	6.768	2.34E-28
Phorbol myristate acetate	Phorbol myristate acetate	Activated	6.662	7.63E-26
SB203580	SB203580	Inhibited	−5.124	1.61E-14
PD98059	PD98059	Inhibited	−4.34	1.17E-15
IL1RN	Interleukin 1 receptor antagonist	Inhibited	−3.874	7.48E-15
SP600125	SP600125	Inhibited	−3.869	1.33E-11
CBX5	Chromobox 5	Inhibited	−3.742	6.92E-06

### IARS2-Activated AKT/MTOR Signaling

**Figure 6:**
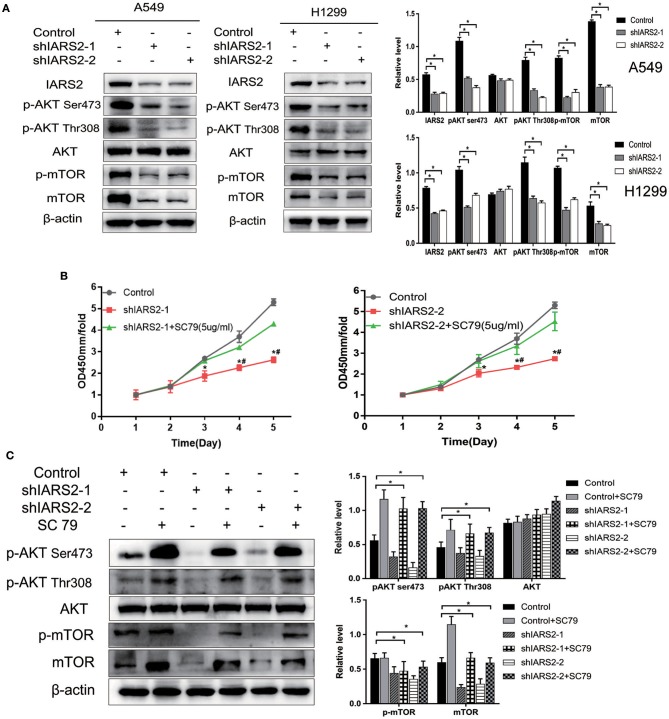
Correlation between IARS2 and AKT/MTOR signaling. **(A)** IARS2 activated AKT/MTOR signaling in A549 and H1299 cells. **(B,C)** The AKT activator SC79 partially restored AKT/MTOR signaling in A549 cells. CCK-8 assays and western blotting were performed to determine protein phosphorylation status after exposure to SC79 (5 μg/mL; 48 and 1 h, respectively). ^*^^, #^*p* < 0.05. (^*^comparison with the control group; #comparison with the applied sc79 group).

We found that the levels of AKT phosphorylation at Ser473 and Thr308 and MTOR phosphorylation in IARS2-silenced A549 and H1299 cells were significantly lower than those in the control group. This indicates that AKT/MTOR signaling pathway activation is significantly inhibited by IARS2 knockdown (Figure 6A, Figure S1).

### AKT Activator SC79 Partially Restored AKT/MTOR Signaling

We treated shIARS2-1- and shIARS2-2-expressing A549 cells with the novel AKT activator SC79, which enhances phosphorylation of all AKT isoforms in a variety of cells. We performed CCK-8 assays and western blotting for protein phosphorylation status after exposure to SC79 (5 μg/mL; 48 and 1 h, respectively). SC79-induced AKT phosphorylation at Ser473 and Thr308, although the induction was slightly weaker than in control cells (Figures 6B,C). SC79 partially restored the IARS2 silencing-induced inhibition of lung cancer cell proliferation. Thus, the loss of IARS2 inhibits, at least in part, growth signaling cascades mediated by AKT.

### IARS2 Regulated the Tumorigenic Capacity of Lung Cancer Cells

**Figure 7:**
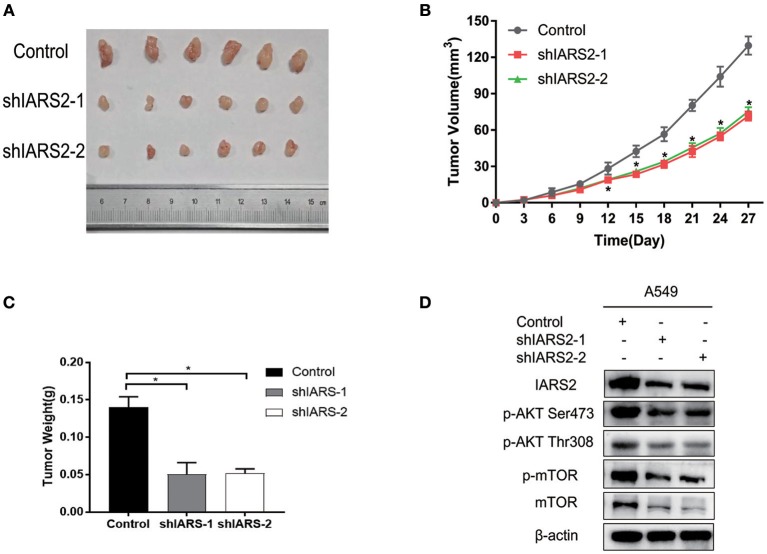
IARS2 silencing inhibits tumor growth *in vivo*. **(A)** Xenografts from mice in each group. **(B)** Tumor volumes were measured at the indicated times. **(C)** Mean weights of tumors obtained from mice. ^*^p < 0.05 compared with the control group. **(D)**Western blotting analyses of protein expression in tumour tissues.

Finally, we assessed the tumorigenicity of IARS2-silenced cells in nude mice. All nude mice developed xenogenic tumors at the injection site (Figure 7A). In mice with IARS2-silenced cells, tumor growth was slower than in the control group (Figure 7B). Similarly, the weights of the excised gene-silenced xenograft tumors were lower than those of the control tumors (Figure 7C). Western blotting analyses of protein expression in tumor tissues (Figure 7D).

## Discussion

Recently, a number of studies have shown that ARSs are associated with multiple tumors and play important roles in triggering or inhibiting tumors, including those associated with stomach, colon, lung, nasopharyngeal, oral, pancreatic, ovarian, prostate, colorectal, and breast cancers (13–22). The mitochondrial enzyme encoded by IARS2 (zone 4, band 1, chromosome 1) is synthesized in the cytoplasm and transported into the mitochondrion where it catalyses the binding of isoleucine to specific tRNAs for the completion of mitochondrial DNA translation. There are few reports on the relationship between IARS2 and disease; its role in tumor development remains unclear. In this study, we found that IARS2 is highly expressed in NSCLC tissues, particularly in adenocarcinoma patients and in tumor T and N stages. Detection of correlations between IARS2 expression and other clinical pathology data, such as gender or age, may require larger studies.

We found that knockdown of IARS2 inhibited the proliferation and clonal formation of A549 and H1299 cells, promoted apoptosis, and induced cell cycle arrest in A549 cells (G0/G1 phase) and H1299 cells (S phase), indicating that it affects DNA and protein synthesis, proliferation, and division in lung cancer cells. IARS2 knockdown significantly inhibits the proliferation and colony formation ability of gastric cancer AGS cells and induces cycle arrest at G2/M phase (10). Together, these results suggest that IARS2 may be a drug target for the treatment of NSCLC.

ARSs play important roles in mitochondrial protein synthesis, thereby contributing to mitochondrial oxidative phosphorylation (23). BCL2, BAX, and caspase 9 are important regulators of mitochondria-dependent apoptosis (24, 25). We found that IARS2 is involved in the mitochondrial apoptosis pathway. Apoptosis, cell cycle arrest, and cell proliferation inhibition are related. Our experimental results consistently indicate that IARS2 plays important roles in the growth, proliferation, and apoptosis of NSCLC cells.

Our cDNA microarray analysis revealed highly significant overlap of 353 IARS2-regulated canonical pathways connected with apoptosis, cancer, cell cycle regulation, and cellular immune responses, and cellular growth, proliferation, and development. Our results indicate that IARS2 is involved in the regulation of a variety of complex biological processes. These data may provide new clues for the study and treatment of lung cancer development, but the detailed mechanisms of IARS2 action require further research.

The AKT/MTOR pathway is a central regulator of cell proliferation, apoptosis, cell cycle, metabolism, and angiogenesis (26). Its activation is associated with tumorigenesis, tumor resistance, invasion, and metastasis. AKT/MTOR signaling pathway-related proteins are also abnormally expressed in liver, lung, breast, bladder, prostate, gastrointestinal, and ovarian cancers (27–33). Aberrant signaling pathway activation is associated with NSCLC and small cell lung cancer cells and cisplatin resistance (34, 35).

Mitochondrial stress leads to increased expression, activation, and nuclear localization of AKT (36). AKT-mediated signaling suppresses mitochondrial oxidation, inhibits apoptosis, and increases cancer cell proliferation (37, 38). Leucyl-tRNA synthetase reportedly initiates mTORC1 activation (39). Both leucyl-tRNA synthetase and IARS are class I ARSs that are presumed to have similar functions in cancer. In this study, IARS2 knockdown promoted mitochondria-dependent apoptosis, suggesting that IARS2 may promote lung cancer cell proliferation and inhibit apoptosis through abnormal activation of AKT/MTOR signaling.

IARS2 knockdown reduced AKT Ser473 and Thr308 and MTOR phosphorylation levels in lung cancer A549 and H1299 cells. Recent work suggested that mitochondrial stress leads to increased expression, activation, and nuclear localization of AKT. The Luo laboratory recently developed the novel AKT activator SC79, which enhances the phosphorylation of all AKT isoforms (40). Using SC79, we demonstrated that AKT activation moderately restored cell proliferation and decreased apoptosis in the absence of IARS.

## Conclusions

In summary, our results indicated that IARS2, an ancient protein synthesis enzyme, may play complex regulatory roles in lung cancer, which should be studied in depth. This study preliminarily revealed the important role of IARS2 in lung cancer pathogenesis and provided a solid hypothetical basis for considering IARS2 expression in the diagnosis and treatment of lung cancer.

## Ethics Statement

This study was approved by the Ethics Committee of the Second Hospital of Jilin University (Changchun, China) and all participants provided written informed consent. The animal experiments were approved by the Institutional Animal Care and Use Committee of Jilin University.

## Author Contributions

KW contributed conception and design of the study. HM, SC, and RW performed the samples collection. JL, CT, and MZ analyzed the data analysis. XD and XJ produced the main draft of the manuscript and made figures. KW and RL obtained funding for the study. All authors contributed to manuscript revision, read and approved the submitted version.

### Conflict of Interest Statement

The authors declare that the research was conducted in the absence of any commercial or financial relationships that could be construed as a potential conflict of interest.

## Supplementary Material

Figure S1 Effect of silencing IARS2 on p-ERK/ERK.
