# Supplementary figures and images for: The Oncogene IARS2 Promotes Non-small Cell Lung Cancer Tumorigenesis by Activating the AKT/MTOR Pathway
Source: Front Oncol. 2019 May 14;9:393. doi: 10.3389/fonc.2019.00393 (PMC6528107; doi:10.3389/fonc.2019.00393)

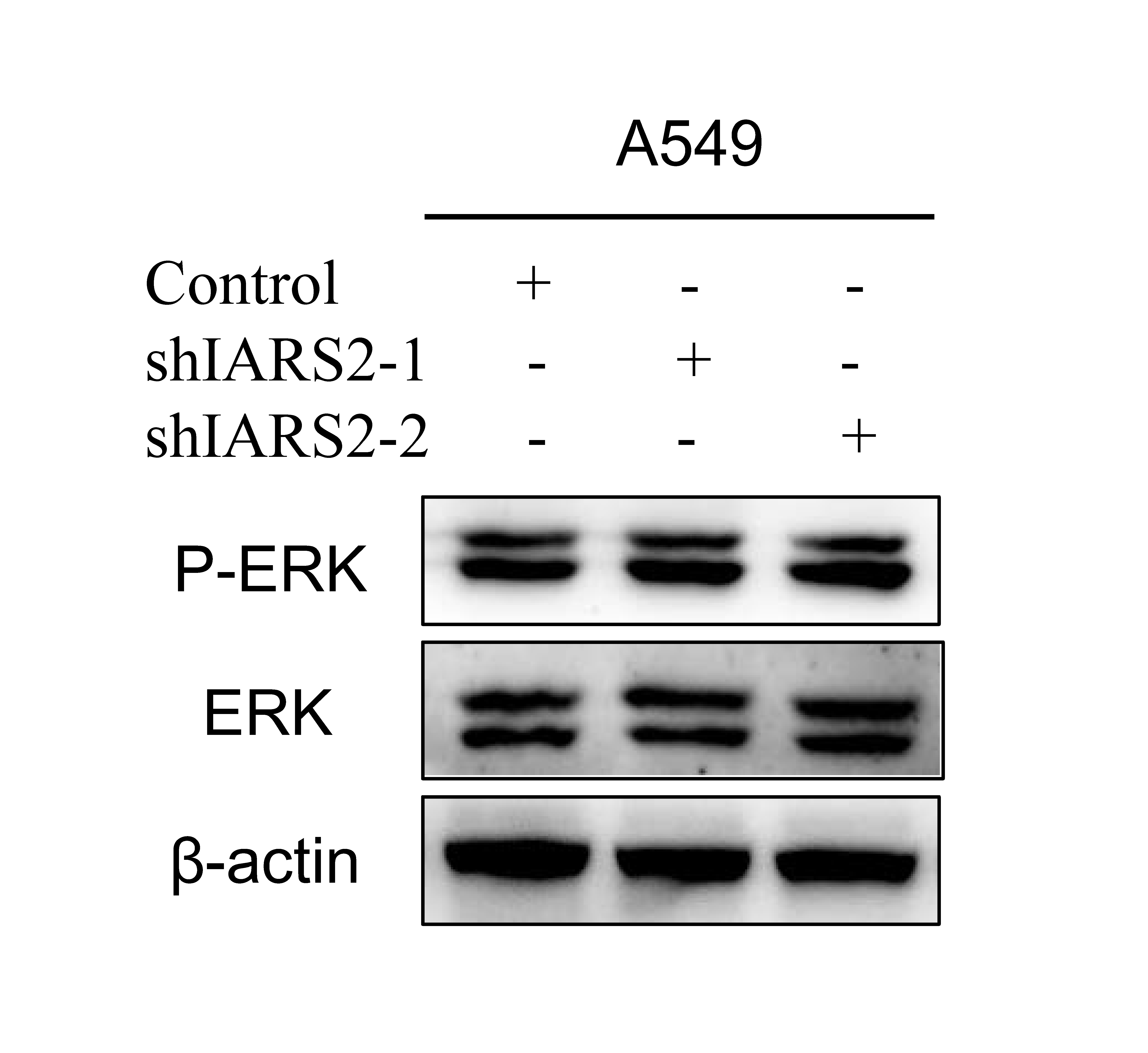

Supplement: Figure S1 — Effect of silencing IARS2 on p-ERK/ERK. [file Image_1.TIF]
